# Presence of Human Papillomavirus DNA in Malignant Neoplasia and Non-Malignant Breast Disease

**DOI:** 10.3390/cimb44080250

**Published:** 2022-08-13

**Authors:** Erika Maldonado-Rodríguez, Marisa Hernández-Barrales, Adrián Reyes-López, Susana Godina-González, Perla I. Gallegos-Flores, Edgar L. Esparza-Ibarra, Irma E. González-Curiel, Jesús Aguayo-Rojas, Adrián López-Saucedo, Gretel Mendoza-Almanza, Jorge L. Ayala-Luján

**Affiliations:** 1 Master in Science and Chemical Technology, Autonomous University of Zacatecas, Zacatecas 98160, Mexico; 2 Academic Unit of Chemical Sciences, Autonomous University of Zacatecas, Zacatecas 98160, Mexico; 3 Academic Unit of Biological Sciences, Autonomous University of Zacatecas, Zacatecas 98068, Mexico; 4 Health Sciences Area, Autonomous University of Zacatecas, Zacatecas 98160, Mexico; 5 National Council of Science and Technology, Autonomous University of Zacatecas, Zacatecas 98000, Mexico

**Keywords:** HPV DNA in breast, nested PCR, breast cancer

## Abstract

Breast cancer is the leading cause of cancer death among women worldwide. Multiple extrinsic and intrinsic factors are associated with this disease’s development. Various research groups worldwide have reported the presence of human papillomavirus (HPV) DNA in samples of malignant breast tumors. Although its role in mammary carcinogenesis is not fully understood, it is known that the HPV genome, once inserted into host cells, has oncogenic capabilities. The present study aimed to detect the presence of HPV DNA in 116 breast tissue biopsies and classify them according to their histology. It was found that 50.9% of the breast biopsies analyzed were malignant neoplasms, of which 74.6% were histologically classified as infiltrating ductal carcinoma. In biopsies with non-malignant breast disease, fibroadenoma was the most common benign neoplasm (39.1%). Detection of HPV DNA was performed through nested PCR using the external primer MY09/11 and the internal primer GP5+/6+. A hybridization assay genotyped HPV. HPV DNA was identified in 20.3% (12/59) of malignant neoplasms and 35% non-malignant breast disease (16/46). It was also detected in 27.3% (3/11) of breast tissue biopsies without alteration. However, there are no statistically significant differences between these groups and the existence of HPV DNA (*p* = 0.2521). Its presence was more frequent in non-malignant alterations than in malignant neoplasias. The most frequent genotypes in the HPV-positive samples were low-risk (LR) HPV-42 followed by high-risk (HR) HPV-31.

## 1. Introduction

Breast cancer is the most common and fatal cancer among women in developed and developing countries. According to data from the World Health Organization, 2,261,419 new breast cancer cases were reported worldwide in 2020, as well as 684,996 deaths [1]. 

It is known that many factors are involved in the development of breast cancer, such as the environment, age, hormones, alcohol consumption, fat in the diet, a diet poor in fruits and vegetables, family history, obesity, tabaquism, alcoholism, number of offspring, breastfeeding, estrogen levels, estrogen receptors [2,3], among others. 

Viruses are considered a controversial etiological risk factor for breast cancer. Viral DNA from human papillomaviruses (HPV), Epstein–Barr virus (EBV), human cytomegalovirus (HCMV), herpes simplex virus (HSV), and human herpesvirus type 8. (HHV-8) has been found in healthy and breast cancer samples [4,5]. However, these results show no pattern, even within the same country, and some are contradictory; moreover, there is no proof of viral breast carcinogenesis [6].

Since Bittner, in 1943, identified the mouse mammary tumor virus (MMTV) as the etiological agent of breast cancer in mice [7], several research groups around the world have been interested in finding a similar relationship between human breast cancer and a viral etiologic agent. In 1995, Wang et al. identified the *env* gene sequence, which codes for the MMTV envelope protein, in 38% of 314 breast neoplasms [8]. In subsequent years, the same research group has worked tirelessly to find a relationship between the onset of breast cancer and infection by the human mammary tumor virus (HMTV). Among other interesting data, they reported the expression of sequences of several proteins from the capsid and envelope of the HMTV virus in ten primary cultures of human breast cancer [9].

In 2017, Islam et al. reported a pattern in the presence of HPV in normal and benign tumors and a markedly increased presence in malignant breast tumors, indicating its pathological importance in breast cancer. HPV was also associated with poor hygienic conditions and patient malnutrition, together with ethnicity [10].

Integrating the HPV genome into the host genome may cause chromosomal instability and trigger carcinogenesis [6,11,12]. Identifying HPV DNA in breast cancer samples suggests the possible role of HPV as a mutagen that promotes breast oncogenesis. However, the prevalence of HPV in breast cancer samples reported by several research groups varies widely, ranging from 0% to 86%. It is often difficult to determine the presence of HPV due to the low viral load in samples or paraffin-embedded tissue, as well as the diversity of techniques employed such as hybridization in situ (HIS), Polymerase Chain Reaction (PCR), Nested PCR, quantitative real-time PCR (RT-qPCR), and Next Generation Sequencing (NGS), among others. Table 1 summarizes the results of HPV DNA found in breast cancer samples worldwide.

Persistent infection with HR-HPV is considered one of the main causative biological factors in developing cervical cancer (CC). HR-HPV 16 and 18 are responsible for more than 65–75% of precancerous cervical lesions and CC. Furthermore, HPV is associated with carcinomas such as head and neck, anal, vulva, oral, vagina, and penile cancer [78]. 

Cervical cancer is the third most common type of malignant tumor and the fourth cause of cancer death among women worldwide. It is also one of the deadliest cancers among women in underdeveloped countries [1]. In Mexico, it is the second-highest cause of cancer death in women due mainly to poor clinical diagnosis in the early stages of the disease and the wide distribution of HR-HPV throughout the country. The first cause of cancer death among women in Mexico and worldwide is breast cancer. For this reason, finding that HPV is an etiological factor for breast cancer would have a high impact on public health programs in Mexico and countries with the highest rates of women mortality from cervical cancer and breast cancer.

However, the oncogenic role of HPV in the development of breast cancer has not yet been clarified, so this study aimed to determine the prevalence of high- and low-risk HPV in breast biopsies diagnosed with benign-alteration and malignant-alteration neoplasms from Mexican women. 

## 2. Materials and Methods

### 2.1. Sample Collection and Classification 

A total of 116 formalin-fixed, paraffin-embedded breast samples from 2009 to 2019 were used for the present study. The remaining tissues were donated to and collected by the Mexican Social Security Institute in Zacatecas. The diagnosis associated with each sample was confirmed by histopathological diagnosis using hematoxylin-eosin (HE) staining and classified according to the World Health Organization (WHO) classification system [1]. The study was approved by the Institutional Ethics Committee of the Autonomous University of Zacatecas and the Mexican Social Security Institute, Zacatecas, and carried out following the guidelines of the Helsinki Declaration. 

### 2.2. Histological Diagnosis

Fresh biopsies were treated with formaldehyde immediately after surgical removal and processed for inclusion in paraffin. Tissue sections were cut and stained with hematoxylin-eosin (HE) for observation under an optical microscope. The pathology specialist performed the analysis and made the histological and clinical diagnoses.

### 2.3. DNA Extraction and Amplification

DNA purification was performed using a QIAmp^®^ FFPE Tissue kit (QIAGEN, Hilden, Germany 56404). Ten 5 µm tissue sections of FFPE breast samples were cut, deparaffinized by incubation with xylene, and washed and rehydrated with ethanol. After complete deparaffinization, the samples were digested with proteinase K at 56 °C for one hour and inactivated at 90 °C. The amount and quality of the DNA were evaluated using a UV-VIS spectrophotometer Q500 (Quawell^®^) at 260–280 nm. The integrity of the extracted DNA and the absence of PCR inhibitors were assessed by polymerase chain reaction (PCR) amplification of the β-globin gene using 5 µM of primers KM29/PCO4 (Table 2) and 50 ng of DNA in a total reaction volume of 25 µL containing: 2.5 µL PCR Buffer (10×, 1.5 µL MgCl_2_ (25 mM), 1 U Taq DNA polymerase (Thermo Fisher Scientific Waltham Massachussetts^®^ EPO402), 0.5 µL of dNTP (10 mM), and water. The amplification of the β-globin gene was performed under the following conditions: initial activation of the enzyme at 95 °C for 2 min, followed by 40 cycles under the following conditions: 95 °C for 30 s, 55.4 °C for 30 s, and 72 °C for 30 s, with a final elongation step at 72 °C for 5 min. The amplicon was visualized in agarose gel (1.5%) stained with ethidium bromide. The images were digitally processed using the Electrophoresis Documentation and Analysis System 120 (Kodak Digital Science).

### 2.4. Detection and Genotyping of HPV

The detection of HPV DNA was first carried out by screening all the samples by end-point PCR using the primers GP5+/6+, which generated a 150 pb fragment. Subsequently, a nested PCR was performed on the samples that tested negative for HPV to increase sensitivity.

Genomic DNA samples from the cervical cancer cell lines SiHa and Caski were used as positive controls for MY09/11 and GP5+/6+ amplification. A paraffin block without tissue and a PCR mix without DNA were negative controls. The primers used are reported in Table 2.

#### 2.4.1. Nested PCR Conditions

For the nested PCR, MY09/11 primers were used to obtain the first amplicon of 450 bp. Subsequently, GP5+/GP6+ primers were used on the first amplicon. The first PCR reaction was carried out with 100 ng of DNA in a total reaction volume of 25 μL containing 2.5 μL of buffer (10×), 1.5 μL of MgCl2 (25 mM), 0.5 μL of each MY09/MY11 primer (10 μM) (Table 2), 0.5 μL of dNTPs (10 mM), 0.25 μL of Taq polymerase (5U/μL) (Thermo^®^, EPO402), and water. The amplification was performed under the following conditions: initial activation of the enzyme at 95 °C for 3 min, followed by 39 cycles under the following conditions: 95 °C for 30 s, 57 °C for 30 s, and 72 °C for 45 s with a final elongation step at 72 °C for 5 min. The second PCR reaction was performed with 5 µL of the first amplicon and the GP5+/6+ primers. The initial activation of the enzyme was performed at 95 °C for 3 min, followed by 39 cycles under the following conditions: 95 °C for 30 s, 48 °C for 30 s, and 72 °C for 30 s with an elongation step at 72 °C for 5 min. The amplicon products were visualized in agarose gel (2%) stained with ethidium bromide.

#### 2.4.2. qPCR Conditions

Quantitative PCR was performed when the samples had a DNA concentration lower than 10 ng/ul. The first amplicon was amplified using primers MY09/11 followed by qPCR. The qPCR was carried out in a 7500 Fast Real-Time PCR System (Applied Biosystems Foster City, California™) in a total reaction volume of 25 µL containing 5 µL of the first amplicon, Platinum SYBR Green qPCR SuperMix-VGD (platinum Taq DNA polymerase, SYBR Green I dye, Tris-HCl, KCl, 6 mM MgCl2, 400 µM dNTPs, UDG), 0.5 µL of each the GP5+/6+ primers (10 µM), 0.1 ul of ROX Reference Dye Solution (25 µM), and water. The amplification was performed under the following conditions: 50 °C for 120 s, 95 °C for 120 s, followed by 40 cycles under the following conditions: 95 °C for 15 s, 48.4 °C for 30 s, 60 °C for 30 s. The data were analyzed using Applied Biosystems 7500 Software v2.0.6, Foster City, California.

#### 2.4.3. HPV Genotyping

Samples positive for amplified HPV L1 gene DNA were subjected to genotyping using the LCD-Array HPV-Type 3.5 kit (Chipron GmbH, Berlin, Germany), which allows for the identification of 32 low-, intermediate-, and high-risk HPV types (6, 11, 16, 18, 31, 33, 35, 39, 42, 44, 45, 51, 52, 53, 54, 56, 58, 59, 61, 62, 66, 67, 68, 70, 72, 73, 81, 82, 83, 84, 91, and 91).

The amplicons generated previously from the biotinylated primers were used for hybridization on the chip: (1) MY09/MY11, which generated a fragment of approximately 450 bp. (2) “125” primers, an internal sequence of the 450 bp fragment (the kit’s own). Both amplicons were combined before hybridization. The PCR was performed according to the manufacturer’s protocol. The reaction mixture was prepared using 2.5 μL buffer (10×), 2 μL MgCl_2_ (25 mM), 1 μL primer mix MY09/MY11 or 2 μL primer mix 125, 1 μL dNTPs (10 mM), 0.3 μL of Taq polymerase (5 U/μL) (Thermo^®^, EPO402), 100 ng of template, and water. Genomic DNA from the CaSki cell line was used as a positive control. The run was conducted as follows: 3 min at 95 °C, followed by 41 cycles of 1 min at 94 °C in denaturation, 1.5 min at 45 °C in alignment, and 1.5 min at 72 °C in extension, and a final extension of 3 min at 72 °C.

Hybridization was carried out according to the manufacturer’s protocol. Biotin-labeled PCR products were hybridized with HPV subtype-specific capture probes immobilized on the surface of the LCD chip. After washing, each field was incubated with a secondary solution (enzyme conjugate). The PCR fragments were then hybridized with capture probes, and the place where they joined was revealed with an enzyme substrate that generated a blue precipitate. Data reading was performed using the LCD SlideReader V9 software.

### 2.5. Statistical Analysis

The breast samples were grouped according to the histological diagnosis. The group measured the presence/absence distribution of HPV DNA by simple counting. Chi-squared tests and Fisher’s exact test were used to compare the presence/absence of HPV DNA between histological diagnosis, sex of patients, tumor size (TMN), and clinical stage. SBR scales were compared using Mann–Whitney tests. The age of patients and tumor size (in cm) between HPV-positive and -negative samples were compared using t-student and Mann–Whitney tests, respectively. All statistical tests were performed in GraphPad Prism version 6. Differences were considered significant when the *p*-value was less than 0.05.

## 3. Results

### 3.1. Histopathological Diagnosis

**Figure 1:**
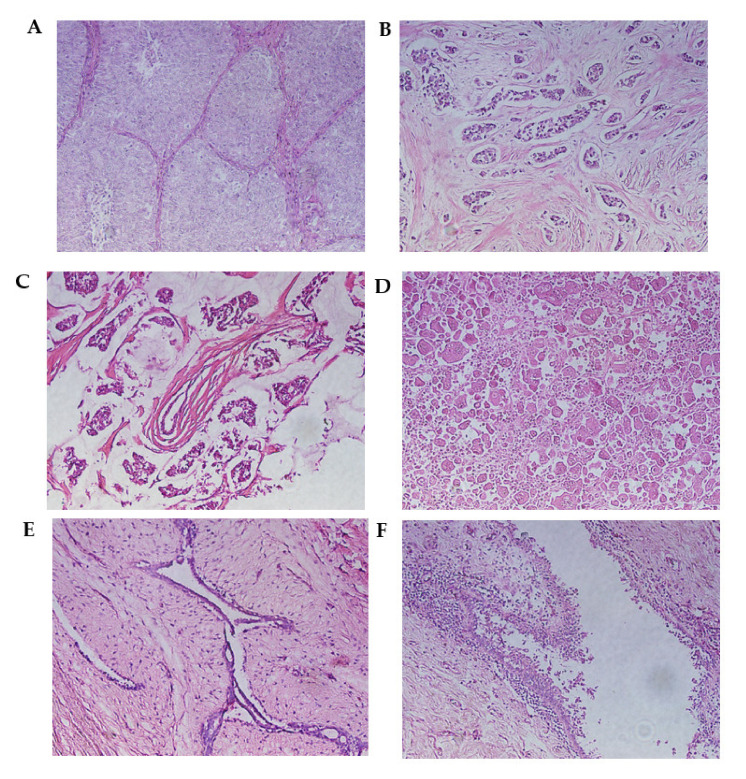
Malignant breast neoplasms. (**A**) Infiltrating ductal carcinoma (solid pattern) separated by connective tissue septa. (**B**) Infiltrating lobular carcinoma. This is characterized by the invasion of the stroma in the form of fine cell cords, called Indian row cords. (**C**) Mucinous carcinoma. Tumor cells are seen within lakes of mucin. (**D**) Metaplastic carcinoma. (**E**) Fibroadenoma. The proliferation of cells can be observed, creating well-defined borders concerning the surrounding normal tissue. (**F**) Mastopathy with hyperplasia. A duct with apocrine metaplasia and foci of hyperplasia can be observed. Hematoxylin and eosin staining. Optical microscopy magnification 20×.

The histopathological diagnosis results from 116 breast samples were classified as shown in Figure 1 and Table 3.

Of all the samples analyzed, 50.9% (59/116) were malignant neoplasms, 4.3% (5/116) were in situ neoplasms, 0.9% (1/116) were borderline neoplasms, and 17.2% (20/116) were benign neoplasms. Benign or non-neoplastic alterations of the mammary gland were also diagnosed, 21.6% (25/116) of the analyzed tissue samples. Breast tissue samples and axillary lymph nodes without alterations were also found, 9.5% (10/116 and 1/116, respectively) of the total.

The distribution of the types of malignant neoplasms diagnosed was as follows: 74.6% (44/59) infiltrating ductal carcinoma, 13.6% (8/59) infiltrating lobular carcinoma, 1.7% (1/59) mucinous carcinoma, and 1.7% (1/59) metaplastic carcinoma. The distribution of benign neoplasms was as follows: 90% (18/20) fibroadenoma, 5% (1/20) adenomyoepithelioma, and 5% (1/20) intraductal papilloma. Regarding non-neoplastic alterations, 60% (15/25) of the samples were diagnosed with cystic fibrous mastopathy, 24% (6/25) corresponded to mastitis, and 16% (4/25) with hyperplasia.

### 3.2. HPV Presence in Breast Samples

HPV DNA was identified in 20.3% (12/59) of malignant neoplasms and 35% of benign neoplasms (16/46). It was also detected in 27.3% (3/11) of breast tissue biopsies without alteration. It was not detected in in situ neoplasms or borderline neoplasms. Regarding malignant neoplasms, HPV was detected in 18.2% (8/44) of the diagnosed biopsies as infiltrating ductal carcinoma, in 25% (2/8) of the biopsies of infiltrating lobular carcinoma, and was in the only sample diagnosed as mucinous and metaplastic carcinoma. Among benign neoplasms, HPV DNA was only identified in 38.9% (7/18) of the fibroadenoma samples. Regarding benign alterations, HPV was detected in 40% (6/15) of the biopsies with cystic fibrous mastopathy and 50% (3/6) of the biopsies of mastitis (Table 3). No relationship was found between the characteristics of the tumor and the presence of HPV in the sample (Table 4).

The most frequent HPV genotype in the samples was LR-HPV 42, which was identified in 19% (6/31) of the analyzed samples, followed by HR-HPV 31 (4/31, 13%) and HR-HPV 59 (3/31, 10%). LR-HPV 44 and HR-HPV 58 were identified in only 6% (2/31) of samples, and HR-HPV 51 in 3% (1/31). Ten percent (3/31) of all HPV-positive samples had mono-infection, while 16% (5/31) had co-infections. Two samples were positive for two virus genotypes, HPV 58/51 (1/31) and HPV 31/42 (1/31) (Table 5). Three samples were positive for more than two genotypes; the most frequent combinations were HPV 31/59/42 (2/31), followed by HPV 42/31/59/44/58 (1/31). The most frequent co-infection was HPV 42/31 (4/31), regardless of the number of genotypes detected per sample.

## 4. Discussion

Breast and cervical cancer are the leading cause of death for women worldwide, mainly in developing countries [1]. HPV is estimated to be associated with more than 5% of all types of carcinomas in humans. High-risk HPV infection has been recognized as an essential factor in developing cervical cancer. It has also been associated with 99.7% of cases of cervical cancer, 50% of head and neck squamous cell carcinomas, and 25% of oropharyngeal cancer [78]. Integration of HPV DNA into the host cell genome is critical in HPV-mediated carcinogenesis, leading to abnormal cell proliferation and malignant progression [10].

In breast cancer, HPV has been proposed in several studies as a probable causative agent of breast cancer carcinogenesis [4,6].

A controversial fact is that HPV DNA has been reported in healthy breast samples. It would be very interesting to make a follow-up study on samples donated by women with healthy breast tissue but positive for HPV DNA to observe if, over the years, they developed some mammary carcinoma, which would support the hypothesis that HPV is an oncogenic factor in breast cancer (Table 1).

The role of HPV in breast cancer carcinogenesis remains controversial due to inconsistent data on the presence of HPV DNA in tumor samples from patients with breast cancer and a lack of clarity regarding the route of HPV transmission from one organ to the other.

The variability of the reported results within the same country could be explained by the quantity and quality of the samples analyzed, considering that breast cancer samples have a lower viral load, making HPV challenging to detect. Other factors that may introduce noise in the study of this subject include the preprocessing of the examined samples, the HPV DNA detection method, and the distribution of HPV among women in each country.

Our results show the presence of HPV DNA in 26.7% (31/116) of the samples, which is in accordance with the findings of other authors in different Latin American countries, whose detection rate ranges from 0 to 49% and the average frequency is 25% [21,27,39,44,54,64] (Table 1). There is a wide range of distribution of genotypes in breast tissue depending on the geographical region. Previous studies in Mexico identified HPV-16, 18, and 33 [35,36,38,43]. Regarding Table 1, it was determined that the five most common genotypes in breast tissue in decreasing order of prevalence are HPV-16, 33, 11, 18, and 6. Similar to the present work, studies conducted in Venezuela and Brazil identified high-risk genotypes HPV-31 and 51 (54,64).

Classified according to their oncogenic characteristics, the prevalence of high-risk HPV types was higher than those with low risk. However, none of these genotypes were identified in this study, and the prevalence of low-risk genotypes was higher than high-risk genotypes. Table 6.

Methodological diversity may partly explain the differences in HPV positivity between studies. However, more importantly, it has been suggested that the viral load of HPV in breast cancer is low [79]. Once cell transformation occurs, viral replication stops, and integration of the viral genome into the host occurs [80]. Under these circumstances, the number of HPV copies decreases sharply. It has been shown that after genome integration, HPV replication decreases; therefore, the choice of detection method and its sensitivity are essential factors to consider since they influence the HPV detection rate [81].

The low prevalence of HPV reported by some studies results from low sensitivity. Therefore, the present study used two variants of the PCR technique to increase the sensitivity and reduce the risk of false negatives. HPV-specific amplicons were detected in 13.8% (16/116) of samples when analyzed by one-step PCR, while the real-time PCR approach increased the positivity rate to 26.7% (31/116).

The differences in HPV prevalence between studies can also be explained by false-positive results, in which contamination is a crucial factor. The present study followed a strict quality control procedure, and the results showed no signs of cross-contamination.

The use of broad-spectrum primers versus specific primers is somewhat controversial since broad-range primers target the HPV L1 gene sequence that could be lost during the integration of the virus into the host genome [27].

It has been suggested that HPV virions present in paraffin-embedded tissue samples may be destroyed during fixation and sample processing. Therefore, HPV may be difficult to detect in tissues preserved for long periods of storage [82]. Some authors suggest fresh tissues may be associated with a higher HPV detection rate compared to samples embedded in paraffin. However, some studies indicate that the low viral load is not a result of tissue samples’ fixation and paraffin inclusion since higher viral loads have been found in formalin-fixed, paraffin-embedded samples than in fresh-frozen cervical cancer tissue samples [83]. Several studies confirm that the type of high-risk HPV and the stage at which the cervical intraepithelial lesion is diagnosed could be triggering factors in the development of breast cancer. According to Table 1, HPV16 is the most common genotype detected in both benign and breast cancer tumors. Among the different types of carcinomas, invasive ductal carcinoma is the breast carcinoma in which HPV DNA is most commonly found.

In 1999, Henning et al. reported that 46% of women with a history of HPV-16-positive high-grade cervical intraepithelial neoplasia (CIN III) lesions were correlated with both ductal and lobular breast carcinomas [26]. Widschwendter et al. and Damin et al. found that the presence of HPV-16 DNA in breast cancer is more frequent in women with a history of cervical cancer [27,28].

Yasmeen et al. made an important observation about breast cancer behavior and HPV. They reported that HPV16 is frequently present in invasive and metastatic breast cancer and less frequently in in situ breast cancer [84].

In a retrospective study, Atique et al. (2017) reported that the incidence of breast cancer was higher among 800,000 HPV-infected patients than among the non-HPV-infected population [85].

**Figure 2:**
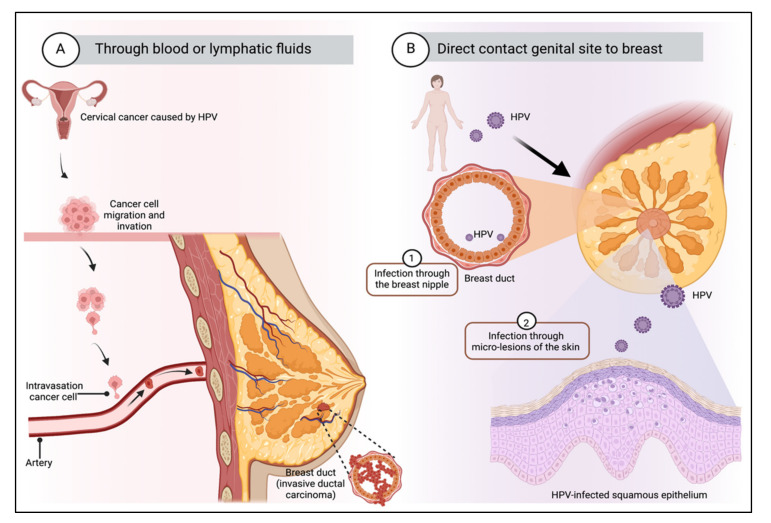
Transmission route of HPV to breast tissue. There are mainly two possible mechanisms: (**A**) Through the blood or lymphatic fluids from the primary site of infection. It is suggested that malignant transformation results from transfection of cells from a primary tumor by plasma flow or that HPV virions can be transported from the site of initial infection to other organs. (**B**) Through the skin of the nipple, by direct contact between the genitals and the breast. The mammary ducts are open ducts and could represent an entry point for virus infection. Transmission can occur through hand contact with the genitals and the mammary gland, which could happen during sexual activity, or through contact of bodily fluids with the fissures of the nipple, which can serve as an entry point for HPV. Created by Biorender.

The mechanism by which HPV can infect mammary gland cells is still unknown. However, two main hypotheses have been proposed; they are summarized in Figure 2. The first one explains that HPV arrives in mammary glands via the lymphatic or blood system through HPV-carrying mononuclear cells present in women with cervical intraepithelial lesions [86]. Other authors conclude that because the HPV life cycle occurs in the epithelial layers, HPV viremia is impossible [43]. The second hypothesis suggests that the mammary gland can be infected with HPV through the skin of the nipple, as demonstrated in the work of Villiers et al. [29], who proposed a retrograde ductal pattern of viral propagation. The exposure of the mammary ducts to the external environment increases the risk of HPV infection since the mammary ducts are open ducts and could serve as an entry point for viral infection. Furthermore, most mammary neoplasms originate from the epithelium of these structures [81]. Sexual transmission is the generally accepted transmission route, although it does not seem to be the only one. Some studies suggest that transmission can occur through hand-mediated contact between the female perineum and the mammary gland, which could occur during sexual activity, or through contact of bodily fluids with nipple fissures, which could serve as an entry point for HPV [4].

**Figure 3:**
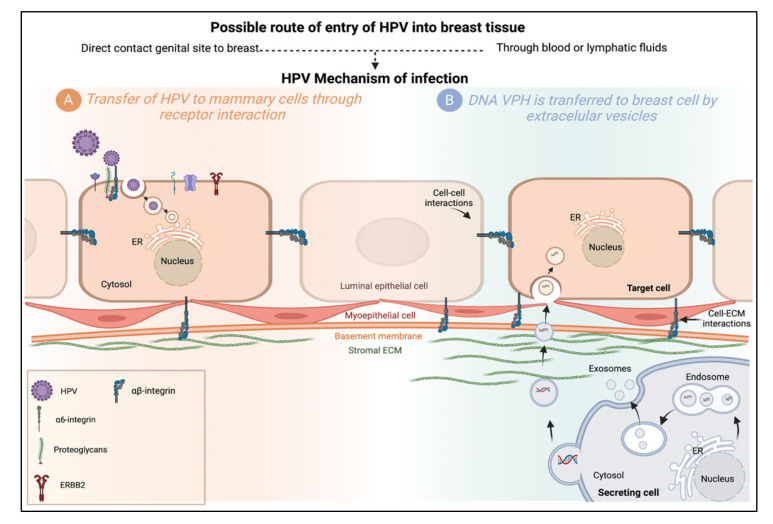
Possible mechanisms of HPV infection in the mammary gland. (**A**) Transfer of HPV to mammary cells through receptor interaction. The HPV-16 capsid interacts with the entry receptor complex composed of growth factor receptors, integrins, and proteoglycans, among others. After HPV binds to this complex, an endocytic process begins. Internalized viruses reside in vesicles directed to acidified multivesicular bodies for capsid disassembly. Viral genomes are transported to the TGN, ER, or core. (**B**) DNA HPV is transferred to breast cells by extracellular vesicles. Transfer of HPV DNA to cells lacking the HPV receptor could be carried out by extracellular vesicles (EVs), microvesicles (EVs), exosomes (Exos), or apoptotic bodies (ABs), which serve as vehicles for cell communication. Cell-to-cell, from a primary site of infection through the transfer of bioactive molecules (proteins, lipids, and nucleic acids). Extracellular vesicles produced from a secretory cell may be internalized by fusion, endocytosis, or phagocytosis, or interact with target cell membrane proteins. Created by Biorender.

Once they have managed to approach the mammary gland cells, the next question is how do HPV viruses penetrate the cells? One hypothesis explains that oncogenic HPV types of the alpha genus use a complex network of proteins for endocytosis and cellular transport, the latter organized by a specific subset of tetraspanins, annexins, and associated proteins such as integrins and EGFRs [87]. Integrins are the extracellular matrix’s central receptors and participate in cell–cell interactions [88]. The α6 integrin has been proposed as the main receptor for HPV-16 in cervical cells [43,89]. In breast tissue, α6 integrins are essential molecules that regulate the growth and differentiation of epithelial cells. Their ability to promote cell anchoring, proliferation, survival, migration, and the activation of extracellular matrix-degrading enzymes suggests that they play an essential role in normal mammary morphogenesis and indicates their potential as HPV receptors and tumor progression promoters [43,88]. Another hypothesis is based on the activity of the extracellular vesicles, including exosomes (Exos), microvesicles (MV), and apoptotic bodies (AB), that are released into biofluids by virtually all live cells (Figure 3).

In 2019, de Carolis et al. detected the same HPV genotype in the same patient’s extracellular vesicles from serum, breast, and cervical tissue. Therefore, the authors suggested that HPV DNA was associated with mammary malignancies and was transferred to the stromal cells of the gland by extracellular vesicles [71].

In an in vitro study, the MCF-10A cell line was transfected with HPV-18 to determine whether HPV may be the starting point of carcinogenesis in breast cancer through APOBEC3B (A3B) overexpression. Their results demonstrated that HPV infection induces upregulation of A3B mRNA and that infected cells exhibited a more malignant phenotype than parental cells since A3B overexpression caused γH2AX foci formation and DNA breakage. The expression of these malignant phenotypes was restricted by shRNA to HPV E6, E7, and A3B. These results suggest an active involvement of HPV in the early stage of breast cancer carcinogenesis through the induction of A3B [90]. A3B expression levels have been reported to be low in most healthy tissues; however, Vieira et al. [91] demonstrated that the E6 protein induces upregulation of A3B RNA in high-risk HPV. It was later observed that in samples from patients with head and neck cancer, there was an overexpression of A3B in HPV-positive tumors.

## 5. Conclusions

The presence of HPV in breast tissue is an important finding, but it is not a sufficient condition to establish an etiological role for this virus in developing breast cancer or any other breast pathology. However, the results suggest a possible role of HPV in breast pathologies as a co-participant in molecular pathogenesis processes that differ from other HPV-associated neoplasms.

According to the results reported in this work, it was possible to detect HPV in breast tissue with malignant neoplasms but also normal tissue. The possible mechanisms by which HPV is present in the breast tissue that would respond have been proposed for its presence in healthy tissue. However, still, there is a pending question. Is it possible that persistent HPV infections in the mammary gland could achieve carcinogenic processes as in the case of cervical cancer?

Since 1992, HPV infection has been proposed as a possible risk factor for the development of breast cancer. Several authors have suggested that the increased incidence of HPV infection may be linked to environmental factors. These observations support the hypothesis of a possible infectious etiology in the development of sporadic breast cancer based on studies that report the presence of sequences of different types of high-risk HPV (oncogenic) in breast carcinoma tissues. However, to date, the results have been controversial and inconclusive. Further studies are required to demonstrate an association between HPV and breast cancer.

## Figures and Tables

**Table 1:** HPV DNA found in breast cancer samples worldwide.

Country	Sample	Method	Control/+HPV	Cases/+HPV	Breast Pathology Predominant	VPH Predominant	Reference
UK	FST	PCR (L1, E7), SB	NS	80/0	IC	NS	[13]
USA	PET	PCR (E6), DB	15/0	28/0	PC	NS	[14]
India	PET	PCR (E6, URR), SB	NS	30/0	IDC	NS	[15]
Austria	PET	PCR (L1), DB	NS	20/0	PD	NS	[16]
Switzerland	PET	PCR (L1)	NS	81/0	IC	NS	[17]
France	PET	PCR (L1)	NS	50/0	IDC	NS	[18]
Tunisia	PET/FST	PCR (L1, E1, E6, E7), ISH	NS	123/0	IDC	NS	[19]
India	FST	PCR (L1, E6, E7)	NS	228/0	IDC	NS	[20]
Brazil	PET	PCR (L1)	NS	79/0	IDC	NS	[21]
China	FroST	PCR (L1)	77/0	77/0	IDC	NS	[22]
Spain	PET	PCR (L1), DEIA	2/0	76/0	IDC	NS	[23]
Greece	FroST	MA (E1)	NS	201/0	IDC	NS	[24]
Italy	PET	PCR (L1,E6), ISH	NS	40/12, 12/0	IC	16	[25]
Norway	PET	PCR (L1,E6)	NS	41/19	IC	16	[26]
Brazil	PET	PCR (E6)	41/0	101/25	IC	16	[27]
Australia	PET	PCR (L1)	NS	11/7	IC	16	[28]
USA	PET	PCR (L1), SEQ, ISH	NS	29/25	IC	11	[29]
Australia	FST	PCR (E6), SEQ	NS	50/24	IDC	18	[30]
Greece	FroST	PCR (L1,E6,E4) RFLP	NS	107/17	IDC	16	[31]
Turkey	FST	PCR (L1, E6,E7)	50/16	50/37	IDC	18	[32]
Syria	PET	PCR (E1), TMA	NS	113/69	IC	33	[33]
Japan	PET	PCR (E6)	11/0	124/26	IC	16	[34]
Mexico	PET	PCR, SEQ	40/0	67/3	IDC	16, 18, 31, 33, 6	[35]
Mexico	PET	PCR (L1), SEQ	43/0	51/15	IDC	16	[36]
Australia	PET	PCR (L1), SEQ, ISPCR	17/3	26/8	IDC,	18	[37]
Mexico	PET	PCR (L1), RT-qPCR	NS	70/17	IDC	16	[38]
Chile	PET	PCR (L1), RT-qPCR	NS	46/4	IDC	16	[39]
China	FroTS	PCR (L1), DB, SEQ	46/0	62/4	IC	16	[40]
Australia	FroTS	PCR (L1), ISH, SEQ	NS	54/27	IDC	18	[41]
Iran	PET	PCR (L1), SEQ	41/1	58/1	IDC	16, 18	[42]
Mexico	PET	PCR (L1)	NS	20/8	MBC	16	[43]
Argentina	FST	PCR (L1)	NS	61/16	IDC	11	[44]
China	FST	HCA	37/6	224/48	IC	NS	[45]
Iraq	PET	ISH	24/320/0	129/60	IC	31	[46]
Italy	PET	INNO-LIPPA (L1)	40/0	40/6	IDC	16	[47]
Iran	PET	INNO-LIPPA (L1)	51/7	55/10	IC	16	[48]
China	DS	PCR, MS	50/0	100/2	IDC	18	[49]
Iran	PET	PCR (L1), SEQ	65/0	65/22	IDC	6	[50]
China	PET	PCR (E7), ISH	83/1	169/25	IDC	58	[51]
Australia	FroST	PCR (L1)	10/1	80/13	IDC	NS	[52]
China	PET	PCR (L1), SEQ	92/0	187/3	IDC	16	[53]
Venezuela	FST	INNO-LIPPA (L1)	NS	24/10	IDC	51	[54]
Australia	PET	PCR (L1), SEQ	18/3	28/13	IC	18	[55]
Corea	PET	PCR	NS	123/22	IDC	51	[56]
Pakistan	PET	PCR (L1)	NS	46/8	IDC	16	[57]
Iran	PET	PCR (L1)	NS	84/27	IDC	16	[58]
China	PET	PCR (E6, E7)	NS	76/23	IDC	18	[59]
Spain	PET	PCR (L1)	186/49	251/130	IC	16	[4]
Thailand	PET	PCR (L1)	350/10	350/15	IDC	16	[60]
India	FST	PCR (L1)	21/2	313/203	IDC	16	[10]
UK	FST	PCR (L1)	36/11	74/35	IC	16	[61]
China	FST	HCA	NS	81/14	IDC	NS	[62]
Pakistan	PET	PCR (L1)	NS	250/45	IDC	NS	[63]
Brazil	PET	PCR (L1)	95/15	103/51	NS	6/11	[64]
Iran	PET	PCR (L1), MA	NS	72/4	IDC	NS	[65]
Morocco	FroST	TS-MPG	12/1	76/19	IDC	11	[66]
Rwuanda	PET	PCR (L1)	NS	47/22	IDC	16	[67]
Denmark	PET	PCR (E6, E7), RH	100/3	93/1	IDC	16	[68]
Iran	PET	RT-qPCR (L1)	40/0	98/8	NS	16,18	[69]
Iran	FroST	PCR (L1, E7)	31/5	72/35	IDC	18	[70]
Italy	PET	PCR (L1), ISH, MS	NS	273/80	IC	16	[71]
USA	PET	PCR (L1), MA	27/8	18/8	IP	11	[72]
Egypt	FroST	RT-qPCR (E6)	15/0	20/4	IDC	16	[73]
Qatar	FST	TS-MPG	50/4	50/10	IDC	16, 35	[74]
Egypt	PET, FST	PCR (L1)	30/0	80/33	IDC	NS	[75]
Sudan	PET	PCR	NS	150/13	NS	16	[76]
Qatar	PET	PCR (E6,E7)	NS	74/48	IDC	52	[77]

Paraffin-embedded tissue: PET; Fresh samples tissue: FST; Frozen samples tissue: FroST; Polymerase Chain Reaction: PCR; In Situ Hybridization: ISH; Tissue Microarray: TMA; Hybrid Capture Assay: HCA; Type-Specific Polymerase Chain Reaction bead-based multiplex genotyping assay: TS-MPG; Microarray: MA; Quantitative Real-Time Polymerase Chain Reaction: RT-qPCR; Sequencing: SEQ; In situ PCR: ISPCR; Dot blot hybridization: DB; Reverse hybridization: RH; Diverse samples (blood, cancer tissue, axillary lymph nodes, normal tissue): DS; Mass spectrometry: MS; Intraductal papilloma: IP; Southern blot: SB; Papillary carcinoma: PC; Paget’s disease: PD; DNA enzyme immunoassay: DEIA; Restriction fragment length polymorphism: RFLP; Upstream Regulatory Region: URR; Not Specified: NS; Invasive Carcinoma: IC; Invasive ductal carcinoma: IDC; Metaplasia breast carcinoma: MBC; Human Papillomavirus protein L1: L1.

**Table 2:** Primers used to amplify ß globin fragment and L1 VPH fragment.

Primer	Sequence 5′-3′	Gene Fragment	Size (pb)
KM29	GGTTGGCCAATCTACTCCCAGG	β-globin	205
PCO4	CAACTTCATCCACGTTACCC	β-globin	205
MY09 *	CGTCCMARRGGAWACTGATC	L1 VPH	450
MY11 *	GCMCAGGGWCATAAYAATGG	L1 VPH	450
GP5+	TTTGTTACTGTGGTAGATACTAC	L1 VPH	140–150
GP6+	GAAAAATAAACTGTAAATCATATTC	L1 VPH	140–150

* M = A + C, W = A + T, Y = C + T, R = A + G.

**Table 3:** Histopathological classification according to World Health Organization.

Classification	n	HPV+	HPV−	% HPV+
**Normal mammary tissue**	**11**	**3**	**8**	**27.3**
Normal breast tissue	10	2	8	20
Normal mammary lymph node	1	1	0	100
**Malignant neoplasm**	**59**	**12**	**47**	**20.3**
Infiltrating ductal carcinoma	44	8	36	18.2
Infiltrating lobular carcinoma	8	2	6	25
**Mucinous carcinom**	1	1	0	100
Ductal carcinoma in situ	5	0	5	0
Metaplastic carcinoma	1	1	0	100
**Non-cancerous breast disease**	**46**	**16**	**30**	**34.8**
*Phyllodes tumors*	1	0	1	0
Fibroadenoma	18	7	11	38.9
Adenomyoepithelioma	1	0	1	0
Intraductal papilloma	1	0	1	0
Hyperplasia	4	0	4	0
Mastitis	6	3	3	50
Fibrocystic mastopathy	15	6	9	40
Total	**116**	**31**	**85**	**26.7%**

% HPV+ per classification; number of samples: n.

**Table 4:** Relationship between HPV and clinicopathological parameters of breast biopsies.

		VPH		*p* Value
		Positive	Negative	
	n (%)	n (%)	n (%)	
Number of samples	116 (100)	31 (26.7)	85 (73.3)	
**Sex**				0.4648 ^a^
Male	2 (100)	1 (50)	1 (50)	
Female	114 (100)	30 (26.3)	84 (73.7)	
**Age (years)**	48.9 ± 13.1	46.9 ± 14.4	49.8 ± 12.6	0.4044 ^c^
CI 95%	(45.8–52.1)	(40.4–53.5)	(46.2–53.4)	
Range	(17–76)	(17–74)	(18–76)	
Malignant neoplasm	59 (100)	12 (20.3)	47 (79.7)	0.2521 ^b^
Non-cancerous breast disease	46 (100)	16 (34.8)	30 (65.2)	
Normal breast	11 (100)	3 (27.3)	8 (72.7)	
**Tumor size (cm)**	3.5 (16.0–1.0)	3.5 (8.0–1.2)	3.7 (16.0–1.0)	0.6788 ^d^
CI 95%	(3.0–4.0)	(2.0–7.8)	(3.0–4.0)	
**Tumor size (TNM)**				0.4422 ^b^
T1 (≤2 cm)	11 (100)	3 (27.3)	(72.7)	
T2 (>2 cm–5 cm)	28 (100)	6 (21.4)	22 (78.6)	
T3 (>5 cm)	6 (100)	2 (33.3)	4 (66.7)	
Not available	9	1	8	
**Clinique stage**				0.6242 ^a^
EII	39 (100)	9 (23.1)	30 (76.9)	
EIII	6 (100)	2 (33.3)	4 (66.7)	
Not available	9	1	8	
**Scale SBR**	8 (9–4)	8 (9–4)	8 (9–6)	0.2470 ^d^
CI 95%	(8–9)	6–9	8–9	
**SBR**				
3–5: Stage I (well differentiated)	2 (100)	2 (100)	0	U
6–7: Stage II (moderately differentiated)	11 (100)	2 (18.2)	9 (81.8)	U
8–9: Stage III (poorly differentiated)	41 (100)	8 (19.5)	33 (80.5)	U

The Scarff–Bloom–Richardson grade: SBR; Primary Tumor, Regional Lymph Nodes, Distant Metastasis: TNM; number of samples: n; centimeter: cm; Confidence Interval: CI; Probability value: *p*; Not significant *p*-value > 0.05; Significant *p*-value < 0.05; *p* = probability value *p* < 0.05; ^a^ Fisher test; ^b^ Pearson test; ^c^ Unpaired Student’s *t* test; ^d^ Mann–Whitney U test; undeterminated: U.

**Table 5:** Genotyping of VHP in FFPE breast samples.

	Histological Type	HPV Genotypes Detected
**Single genotype** **n = 3 (10%)**	Mucinous carcinoma	44
	Fibroadenoma	42
	Mastitis	42
**Multiple genotype** **n = 5 (16%)**	Fibroadenoma	58 + 51
	Fibroadenoma	31 + 42 + 59
	Cystic fibrous mastopathy	31 + 59 + 42
	Cystic fibrous mastopathy	31 + 42
	Mastitis	42 + 31 + 59 + 44 + 58

number of samples: n.

**Table 6:** Comparative table of HPV reported in breast tissue samples in Latin America.

Country	Sample	Method	Cases/+HPV (%)	HPV Genotype						Reference
Venezuela	FST	PCR (L1), RH	24/10 (41.7)	51	18	33	6	11		[54]
Brazil	PET	PCR (L1), TS-MPG, SEQ	103/51 (49.5)	6	11	18	31	33	52	[64]
Brazil	PET	PCR (E6)	101/25 (24.8)	16	18					[27]
Brazil	PET	PCR (L1)	79/0 (0)							[21]
Argentina	FST	PCR (L1), RT-qPCR, SEQ	61/16 (26.2)	11	16					[44]
Chile	PET	PCR (L1), RT-qPCR	46/4 (8.7)	16						[39]
Mexico	PET	PCR (E1), SEQ	67/3 (4.5)	16	18	33				[35]
Mexico	PET	PCR (L1), SEQ	51/15 (29.4)	16	18					[36]
Mexico	PET	PCR (L1), RT-qPCR	70/17 (24.3)	16	33					[38]
Mexico	PET	PCR (L1), RT-qPCR	20/8 (40)	16	18					[43]
Mexico	PET	PCR (L1), MA	116/31 (26.7)	42	31	59	58	44	51	Present study

Paraffin-embedded tissue: PET; Fresh samples tissue: FST; Polymerase Chain Reaction: PCR; Type-Specific Polymerase Chain Reaction bead-based multiplex genotyping assay: TS-MPG; Microarray: MA; Quantitative Real-Time Polymerase Chain Reaction: RT-qPCR; Sequencing: SEQ; Reverse hybridization: RH; Human Papillomavirus protein L1: L1.

## Data Availability

Not applicable.
